# Study protocol for developing deprescribing clinical practice guidelines: evidence-based GRADE methodology and a Delphi consensus method

**DOI:** 10.1186/s12877-025-06202-2

**Published:** 2025-07-19

**Authors:** Hui Wen Quek, Amy Page, Kenneth Lee, Christopher Etherton-Beer

**Affiliations:** 1 Centre for Optimisation of Medicines, School of Allied Health, The University of Western Australia, 35 Stirling Hwy, Crawley, Western Australia 6009 Australia; 2 Western Australian Centre for Health and Ageing, The University of Western Australia and Royal Perth Hospital, Perth, Australia

**Keywords:** Aged, Delphi technique, Drug utilization, Geriatric medicine, Clinical decision-making

## Abstract

**Background:**

Deprescribing has emerged as a strategy to reduce the use of potentially inappropriate medicines, particularly in older people. Evidence-based deprescribing clinical practice guidelines are a key enabler in integrating deprescribing into routine care. This protocol outlines the development of deprescribing clinical practice guidelines targeting many commonly prescribed medicines for older people, specifically focusing on applying the evidence-based Grading of Recommendations Assessment, Development, and Evaluation (GRADE) methodology alongside a Delphi consensus-building process.

**Methods:**

The guideline development process follows the World Health Organisation Handbook for Guideline Development, Australian National Health and Medical Research Council Guideline Development Methodology, and the Appraisal of Guidelines for Research & Evaluation (AGREE) II Instrument with adaptations made to suit the guideline’s purpose, available resources, and the urgent need for recommendations to support clinical decision-making. This project is developed by a multidisciplinary healthcare team, representatives from professional organisations, and patient or carer stakeholders. The development involves a two-part sequential approach: evidence-deriving using a structured GRADE methodology and consensus-building processes using a standardised Delphi approach. Firstly, a comprehensive systematic review and meta-analysis of the literature was conducted to identify evidence related to deprescribing in older people, with the evidence presented and certainty assessed using the GRADE framework. Where quality evidence is available, evidence-based recommendations will be formulated following the evidence-to-decision GRADE framework. For areas with insufficient quality evidence, consensus-based recommendations will be developed using a modified Delphi method. Additional good practice statements will be developed where necessary to facilitate the practical application of these recommendations.

**Discussion:**

Given the large scope of the currently proposed guidelines, the proposed approach discussed in this protocol is adapted based on several important considerations on the practical, operational, and resource issues. Given deprescribing is an emerging area and the limited availability of evidence for some drug classes, expert consensus and input from patient representatives offer a valuable alternative for recommendation development. The final guideline will provide clinicians with broad guidance for deprescribing common medicines used in older people that complement existing single-drug-class deprescribing guidelines and other treatment guidelines.

**Trial Registration:**

Not applicable.

## Introduction

### Background

The ageing population presents a unique set of challenges that necessitate a careful approach to medication management. Older people are more likely to have comorbidities that prompt the use of multiple medicines to manage their complex medical conditions. As such, polypharmacy, defined as the concurrent use of five or more medicines, is prevalent in older people [1, 2]. A systematic review revealed that up to 93% of people aged 65 and over globally experience multimorbidity, with polypharmacy affecting as many as 87% of this population [3]. While polypharmacy has been associated with negative outcomes including falls, frailty, and mortality [4–6], the number of medicines does not necessarily indicate the appropriateness of a medication regimen [7, 8]. Medicines can play a crucial role in preventing future complications and providing symptomatic relief, thereby significantly enhancing a person’s functioning and quality of life. Consequently, it is essential to distinguish between appropriate and inappropriate polypharmacy by applying careful clinical judgment. Inappropriate polypharmacy increases the risks of adverse drug events, medication non-adherence, hospitalisations, geriatric syndromes, and mortality [9, 10]. Older people, in particular, are more vulnerable to these negative consequences of potentially inappropriate medicines than younger people due to reduced physiological reserves. Medication optimisation is a process to ensure safe and effective use of medicines [11] and deprescribing forms a part of the process.

Deprescribing is a systematic process of tapering, stopping, discontinuing, or withdrawing one or more medicines with the goal of managing inappropriate polypharmacy and achieving improved outcomes [12–14]. Deprescribing has emerged as a critical component of patient-centred care and is viewed as an effective intervention to reduce the use of potentially inappropriate medicines [15, 16]. While deprescribing has been extensively explored in various contexts, its implementation in routine clinical practice has not been widely reported, with healthcare professionals consistently citing a lack of detailed guidance as a barrier to deprescribing [17, 18]. A scoping review indicated that only 29% of existing treatment guidelines incorporated at least one recommendation about deprescribing, with a primary focus on prescribing practice for disease management [19].

Evidence-based deprescribing guidelines are seen as a facilitator of deprescribing to bridge the gap between scientific evidence and clinical practice [20–22]. Clinical practice guidelines are ‘systematically developed statements to assist practitioner and patient decisions about appropriate health care for specific clinical circumstances’ [23]. As opposed to guides, clinical practice guidelines are formal documents developed through a rigorous and standardised process that involves systematic reviews of existing evidence and expert consensus. A qualitative study has shown that evidence-based deprescribing guidelines increased clinicians’ perceived self-efficacy in developing and implementing deprescribing plans for certain drug classes [24]. However, clinical practice guidelines for deprescribing currently exist for only a limited number of drug classes. Although more recently, an attempt has been made to develop a comprehensive guideline for common psychiatric medicines, this guide was developed using a different approach than standard clinical practice guidelines, which may require different critical appraisal methods [25]. Additionally, a study shows that deprescribing recommendations currently incorporated in treatment guidelines do not contain clear and actionable recommendations with a substantial variation in their content and format that may further confuse healthcare professionals [19]. Deprescribing is an area of practice requiring complex decision-making in partnership with patients, their carers, and family members. Hence, a specific clinical practice guideline targeting deprescribing may improve effective implementation in clinical practice. Current clinical practice guidelines for deprescribing exist for antipsychotics [26], benzodiazepine receptor agonists [27], proton-pump inhibitors [28], antihyperglycemics [29], opioid analgesics [30], as well as cholinesterase inhibitors and memantine [31]. The population for the systematic review conducted for these guidelines included people aged over 18, with most of the guidelines did not provide specific recommendations for older people. The models of care for older people and their care goals can be vastly different to those of younger people, especially for older people living with frailty [32, 33]. Additionally, single drug class guidelines may have limited application in addressing inappropriate polypharmacy commonly seen in older people.

### Objective

In light of the World Health Organisation (WHO) “Medication Without Harm” global initiative to reduce avoidable medication-related harm [34], recommendations to guide clinical decision-making and to promote judicious deprescribing in practice are the need of the hour. Despite this urgent need, there is a notable lack of deprescribing guidelines for many commonly used medicines. To address this gap, we aim to develop a clinical practice guideline for deprescribing that encompasses medicines frequently prescribed to older people. The current protocol outlines the development of this guideline, specifically focusing on applying the evidence-based Grading of Recommendations Assessment, Development, and Evaluation (GRADE) methodology alongside a Delphi consensus-building process.

## Methods

### Study design

The development of this clinical practice guideline consists of evidence-deriving and consensus-building processes. For the first part, evidence will be derived using a systematic review and meta-analysis of the literature and a rigorous assessment of the certainty of the evidence using the Grading of Recommendations Assessment, Development, and Evaluation (GRADE) framework. Systematic reviews and meta-analyses are considered the gold standard in evidence synthesis [35] whereas the GRADE framework is increasingly seen as the preferred approach for summarising findings in systematic reviews and rating the certainty of a body of evidence [36]. For the second part about the consensus-building process where evidence is insufficient or lacking, a modified Delphi approach will be used as it is well-suited for gathering input from individuals across diverse professions and specialties, especially when there are expected differences in opinions [37, 38]. The traditional Delphi method involves generating qualitative data in the first round of data collection to guide the development of statements for the subsequent rounds. A modified method will be used that omits the first qualitative round and begins the series of rounds with a set of carefully selected statements derived from the literature, previous research, or existing clinical practice guidelines for treatment [39]. The modification was carefully considered to expedite the process, minimise participant fatigue and increase overall engagement throughout the subsequent iterative rounds without compromising the integrity of the consensus-building process [40]. We acknowledge the potential biases inherent in the Delphi approach. Nevertheless, the Delphi technique provides advantages particularly through the anonymity of responses during the survey rounds. This anonymity helps mitigate potential dominance effects, halo effects, and groupthink commonly encountered in other group settings such as a focus group [41]. Additionally, the iterative process allows for controlled feedback, ultimately facilitating the achievement of group consensus. We plan to include a diverse group of Delphi panel members and establish a predetermined cut-off response rate to mitigate potential selection and response biases.

The development of the clinical practice guideline follows the principle of robust clinical guideline development standards as outlined in the WHO Handbook for Guideline Development, Australian National Health and Medical Research Council (NHMRC) Guideline Development Methodology and the Appraisal of Guidelines for Research & Evaluation (AGREE) II Instrument and User’s Manual [42–44]. In Australia, guidelines developed by groups external to NHMRC may be approved by NHMRC. While it is not mandatory for guidelines to obtain approvals, NHMRC offers step-by-step guidance to produce high-quality guidelines. Additionally, multiple tools have been developed for guideline appraisal among which the AGREE II instrument is the most commonly used and forms part of the process suggested by NHMRC [36]. Adhering to the processes detailed in the WHO handbook, NHMRC guidance and AGREE II instrument will ensure the guidelines meet the requirements for methodological rigour in the development and reporting of guidelines. However, developing a new guideline is typically a costly and time-consuming process, which may pose a challenge to resource-constrained settings [45]. We opted for pragmatic design adaptations to suit the purpose of the guideline, and available resources, in consideration of the urgent need for recommendations to support clinical decision-making.

### Purpose of the guideline

The guideline will provide guidance on the key aspects for deprescribing considerations in people aged 65 years and over, which are to determine when, how, and for whom a medicine should be deprescribed, as well as identify monitoring requirements during deprescribing and the ongoing treatment needs as applicable.

### Scope of the guideline

This guideline prioritises providing recommendations for medicines commonly prescribed and dispensed to older people as it is likely to have the largest impact on clinical practice. We leveraged data from the Australian Pharmaceutical Benefits Scheme (PBS) to identify the top 100 medicines as priorities for future deprescribing efforts. The PBS is routine administrative data that provides an accurate representation of common medicines used by the population. The Australian PBS subsidises the cost of most medicines in Australia for eligible residents, with over half (54%) of PBS-subsidised medicines dispensed to people aged 65 and over [46]. This guideline will be limited to medicines intended for regular use. Hence, medicines prescribed for short-term, intermittent, as required, or acute use only (e.g. systemic or topical antibiotics) will not be included.

The guideline steering committee analysed the PBS data for people aged 65 or over who were dispensed PBS-listed medicines in 2023 to identify the top 100 medicines with the highest dispensing volume or the largest number of unique persons dispensed, excluding non-regular medicines. The volume-based metric represents the total number of dispensing in a calendar year, while the person-based metric refers to the number of people who received the medicine in a calendar year. The person-based metric is included to account for medicines with less frequent dosing, such as denosumab, which is typically administered every six months. This methodology was previously adopted in a study investigating the inclusion of information about medication withdrawal and medicine use in older people [47]. While the primary focus of this guideline will be common PBS-listed medicines, the guideline development group will review and consider on a case-by-case basis the inclusion of additional medicines where evidence for deprescribing is identified in the systematic search. The rationale is to not exclude medicines simply because they are not listed on the PBS but to consider the potential risks of inappropriate use and the impact of deprescribing.

### Stakeholder involvement

#### Guideline steering committee

The guideline steering committee (authors of the current protocol) has a primary role of guiding and overseeing the overall development of the guideline. Their responsibilities are to establish a guideline development group, propose the topic and scope of the guidelines to members of the guideline development group, refine the key clinical questions, as well as plan and lead the development of high-quality, credible recommendations and good practice statements. The steering committee will support the implementation of the guideline in clinical practice and actively take part in the dissemination process.

#### Guideline development group

The guideline steering committee will establish a guideline development group with members from each of the following categories: (1) physicians including general practitioners, geriatricians, clinical pharmacologists, and geriatric psychiatrists, (2) nurse practitioners (3) pharmacists, (4) statisticians, (5) policymakers, (6) allied health professionals (optometry, dental, podiatry, physiotherapy, physiotherapist), (7) methodologist with experience in guideline development, methodology or systematic reviews, (8) expert in implementation science or behavioural science, (9) pharmacoepidemiologist, (10) health economist, and (11) patients or carers with lived experience. Clinicians and pharmacists must be practising in the field of geriatric care or pharmacotherapy relevant to people aged 65 and over meeting one or more of the following selection criteria:

1. Demonstrable clinical experience in the field of geriatric and gerontology or specialised in providing pharmaceutical care for older people (e.g. practice-based experts who are practising or having practised in the field for at least five years).
2. Recognised as an expert in the field by peers (e.g. invitation to a relevant symposium, conference or other academic events as a speaker or presenter, or membership in an association or research group).
3. Recent publications as a first or last author on the relevant topic in peer-reviewed journals within the past five years.
4. Post-graduate qualification or current credential relevant to geriatric pharmacotherapeutics (e.g. a geriatrician or a pharmacist credentialed with a certificate in geriatric pharmacy).

At least one member, regardless of profession, will be practising in each hospital, residential aged care facility and private practice settings and at least one member will be practising in a rural or remote area. Individual members may fulfil multiple criteria, such as a general practitioner practising in a hospital in a rural area.

All members of the guideline development group and steering committee will be required to declare any perceived or actual financial or non-financial competing interests. The guideline steering committee will record and manage potential conflicts of interest relevant to the guideline development. Members of the guideline development group will be identified through professional networks and snowball sampling. If there is a lack of relevant content expertise for a specific therapeutic area, the guideline steering committee will be responsible for recruiting additional clinical experts with relevant expertise and credentials based on their existing professional networks.

At least four members of the guideline development group will represent a specific consumer advisory group consisting of at least one layperson, patient, and carer with lived experience. These individuals will be identified through the Western Australian Health Translation Network Consumer and Community Involvement Program. These individuals will be invited to provide critical insights into the challenges and needs that are often overlooked in clinical practice, provide their input on draft deprescribing recommendations, and ensure that at every stage the views of patients and carers are prioritised. Layperson, patients and carers will be reimbursed for their time. By integrating their perspectives, we aim to create guidelines that resonate with the actual experiences and preferences of patients and the wider public, ensuring greater relevance and uptake in real-world settings.

All members of the guideline development group will contribute by reviewing draft recommendations and taking part in the modified Delphi study to establish consensus-based recommendations for common drug classes without evidence from the literature.

#### External experts

External experts will be individuals with the expertise and experience relevant to the methodology or the content of the guideline who have indicated a preference to provide external expert feedback independent from the guideline development group.

### Rigour of guideline development

**Fig. 1:**
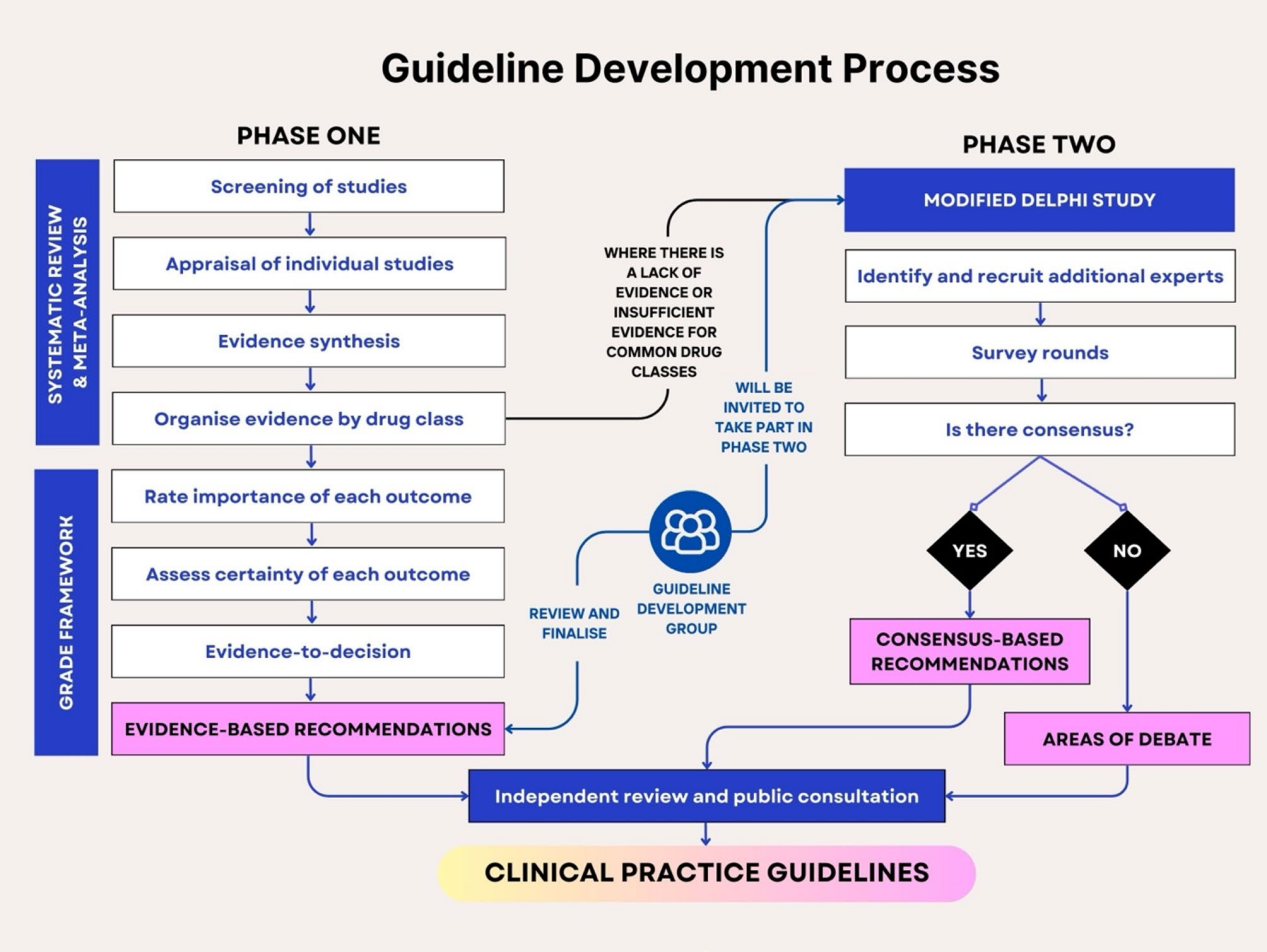
Guideline development process focusing on GRADE Methodology and a Delphi Consensus Method. A two-phase process for developing clinical practice guidelines. Phase One involves systematic review and meta-analysis to synthesise evidence and recommendation development using the GRADE framework. Phase Two addresses areas where evidence is insufficient, inconclusive, or unavailable, aiming to develop consensus-based recommendations using a modified Delphi approach or identifying “Areas of Debate.” The final recommendations will further undergo independent review and public consultation.

The development of the guideline comprises two main phases (Fig. 1). Phase 1 involves synthesising evidence using a systematic review and meta-analysis approach. Phase 2 will involve presenting draft recommendations to the guideline development group to determine consensus using a systematic modified Delphi method.

#### Phase 1: synthesising evidence

##### Identifying relevant evidence

The guideline steering committee updated a 2016 systematic review and meta-analysis [48] assessing the effects of deprescribing in older people to capture new evidence that emerged since the original publication [49]. This systematic review and meta-analysis summarised comprehensive findings on the effects of deprescribing on mortality, physical health, cognitive function, quality of life, effect on medication regimen, and adverse drug withdrawal events. The process follows the Cochrane Handbook for Systematic Reviews of Interventions, which consists of an updated literature search, screening for study inclusion, data extraction, quality appraisal, data analysis and synthesis (meta-analysis), and interpretation of findings [50]. The methods have previously been described in a published protocol [51]. The updated systematic review and meta-analysis has since been published [49]. Briefly, the review had broad selection criteria without limitations on study settings, patient sub-groups, or types of medicines targeted, aiming to capture all relevant studies related to deprescribing in older people. The Population, Intervention, Comparator and Outcomes (PICO) framework guiding the evidence review as well as the predetermined inclusion and exclusion criteria for screening relevant studies are shown in Table 1.

**Table 1:** Population, intervention, comparator and outcomes (PICO) for evidence review

	Description	Inclusion Criteria	Exclusion Criteria
**Population**	Adults aged 65 years and over with *no* limitation placed on setting, cognitive function, or comorbidities	• Aged 65 years and over, defined as studies where one of the following applies: • Mean participant age is ≥ 65 years • Greater than 75% of participants are aged ≥ 65 years • Data from people aged ≥ 65 years can be extracted	Unclear age or studies including only moribund, terminal, or palliative participants
**Intervention**	Deprescribing of medicine(s)	Medicine(s) intended for regular use	Medicine(s) intended for short-term, intermittent, as required, or acute use only
**Comparator**	Continuation of the medicine(s) or no comparator	• Continuation of the medicine(s) • No comparison • Non-pharmacological intervention	Substitution with an alternative medicine(s)
**Outcomes**	• Mortality • Adverse drug withdrawal events • Cognitive function • Quality of life • Other health-related outcomes • Effect on medicine regimen	Clinically relevant health outcomes, significant events or surrogate endpoints	Outcomes of uncertain or limited clinical relevance

Electronic database searches were conducted in CINAHL, Medline, Embase, Scopus, Web of Science, and ProQuest (Dissertations and Theses Global) to identify relevant published studies up to April 2024 in which older people (aged 65 years and older) had at least one medicine deprescribed. Both experimental (randomised or non-randomised controlled trials) and observational studies with or without concurrent control groups (before-and-after, case-control or cohort studies) were included. Studies were grouped by study designs and targeted medicines for data analysis. The risk of bias was assessed using the Cochrane tool and the Newcastle-Ottawa tool. Odds ratios or mean differences were calculated as the effect measures using either the Mantel–Haenszel or generic inverse-variance method with fixed- or random-effects meta-analyses.

##### Formulating draft recommendations

The certainty of the evidence in the systematic review and meta-analysis will be rated using the GRADE approach. This structured and transparent approach will enable recommendations to be synthesised based on the evidence and its certainty, while also considering overall benefits and harms, patient values and preferences, resource implications, and the feasibility of implementation [52]. The GRADE approach has been adopted by national and international organisations as a preferred approach to rate the certainty of evidence in systematic reviews to develop and determine the strength of recommendations in clinical practice guidelines [53]. The key steps in developing recommendations using the GRADE approach are: 1) selecting and rating the importance of outcomes, 2) summarising the evidence, 3) determining the quality of evidence, and 4) moving from evidence to recommendations.

It is acknowledged that the importance of an outcome may only become known once evidence is reviewed, or the analyses were carried out (e.g. serious adverse effect). The search strategy for our systematic review and meta-analysis has thus included broad outcomes of mortality, physical health, cognitive function, quality of life, effect on medication regimen, and adverse drug withdrawal events. Initially, the guideline steering committee will organise the evidence of outcomes identified from the systematic review and meta-analysis by drug classes. Each outcome will be rated on its relative importance by the guideline development group for decision-making: critical, important but not critical, or low importance [54]. Outcomes rated as critical and important will be used to produce the GRADE evidence profile and GRADE summary of findings table which will bear on guideline recommendations.

Two researchers trained in the GRADE approach will independently assess the certainty of the evidence for each outcome by considering eight GRADE criteria (risk of bias, directness of evidence, consistency and precision of results, risk of publication bias, magnitude of the effect, dose-response gradient, and influence of residual plausible confounding). Outcomes will be rated as high, moderate, low, or very low certainty (Table 2), with discrepancies between the researchers resolved through discussion and consensus. These outcomes along with the certainty of the evidence will be included in the GRADE evidence profile and subsequently GRADE summary of findings table. The latter is intended to be a quick summary and will not contain details of the judgments about the certainty of the evidence.

**Table 2:** GRADE certainty of evidence ratings [54]

GRADE ratings	Definitions
⨁⨁⨁⨁ High	We are very confident that the true effect is close to the estimated effect.
⨁⨁⨁◯ Moderate	We are moderately confident in the estimated effect: The true effect is probably close to the estimated effect, but there is a possibility that it is substantially different.
⨁⨁◯◯ Low	We have limited confidence in the effect estimate: The true effect may be substantially different from the estimated effect.
⨁◯◯◯ Very low	We have very little confidence in the effect estimate: The true effect is likely to be substantially different from the estimated effect.

The GRADE Evidence-to-Decision framework provides a structured and transparent framework to develop recommendations based on the relative importance and certainty of the evidence, while also considering the overall benefits and harms, patient values and preferences, implications for resource utilisation, equity, acceptability and feasibility of deprescribing [52]. The guideline steering committee will follow the GRADE Evidence-to-Decision framework to draft recommendations. Specifically, when considering the overall balance of benefits and harms, best estimates of the magnitude of effects on desirable and undesirable outcomes and the relative importance of outcomes based on estimated values and preferences will be considered. Patient values and preferences for each drug class will be investigated based on consultations with the layperson, patient, or carer representatives in the guideline development group as well as non-systematic reviews of the available literature, or clinicians’ experience of interactions with their patients. Investigations on the implications of resources, equity, acceptability and feasibility of deprescribing will rely on non-systematic reviews of the available literature, expert opinions or individual experiences of the guideline development group members.

Draft recommendations will be presented to all members of the guideline development group along with the evidence for review presented in tables and as narrative reviews, and where appropriate including statistical data such as meta-analysis results. The group members will be briefed on the guideline development methodology and the GRADE framework so each member can independently apply their judgement in a consistent and systematic way. The guideline development group will ultimately determine the type of recommendation categorised based on the type and source of evidence that supports them (Table 3). For evidence-based recommendations, the strength (strong or weak) and direction (for or against) of recommendations will also be determined. The guideline steering committee will be responsible for revising the draft recommendations based on feedback from the development group ensuring all appropriate viewpoints are considered. Where the guideline development group identifies there is insufficient quality evidence or lack of evidence, they may choose not to make a recommendation. Alternatively, consensus methods (Phase 2) will be used to develop recommendations or good practice statements grounded in expert opinions and individual patient or carer experiences.

**Table 3:** Types of guideline recommendations

Recommendation types	Description
Evidence-based recommendation	Recommendation developed based on quality and consistent evidence identified from a systematic review and meta-analysis linking deprescribing to outcomes.
Consensus-based recommendation	Recommendation developed by the guideline development group using Delphi consensus methodology when evidence is insufficient, inconclusive, or unavailable, following a systematic review and meta-analysis approach to search for evidence. The purpose of consensus-based recommendation is to fill the knowledge gap.
Good practice statement	An actionable statement developed by the guideline development group using Delphi consensus methodology to support recommendations, or to guide deprescribing processes when there is indirect but high-quality supportive evidence and other criteria for good practice statement development are met [55].

#### Phase 2: consensus-building process using a modified Delphi method

While peer-reviewed evidence has long been considered the gold standard for developing guideline recommendations [56], research is not always available to inform guideline recommendations. As deprescribing is a relatively new field, there is sparse evidence for patient-important outcomes, as shown in previous systematic reviews [48, 49]. In situations where there is insufficient information, consensus methods provide another means of synthesising information grounded in expert opinions and experiences.

For the second phase of guideline development, the guideline steering committee will use surveys to elicit opinions from leading experts and patients or carers to reach a consensus on core recommendations for deprescribing clinical practice guidelines to fill the knowledge gap (Fig. 1). 

##### Selection, identification and recruitment of the panel

All members of the guideline development group involved in Phase 1 will take part in the modified Delphi study in Phase 2 to establish consensus-based recommendations for common drug classes with insufficient or a lack of evidence from the literature review and where appropriate, good practice statements. As the guideline steering committee drafted the recommendations, they will not be involved in the decisions made by the consensus panel. Guideline development group members will be included if they are willing to participate in all Delphi rounds and declare ongoing conflicts of interest. While literature commonly suggests a panel size of 10 to 18 for a Delphi study, we plan to include a minimum of 20 participants to account for potential attrition [57].

##### Delphi method

The online surveys will be administered using the Qualtrics software (Qualtrics, Provo, UT, USA. https://www.qualtrics.com) [58]. The content of the survey will include proposed recommendations or statements that are specific to drug classes and will be organised into the pre-defined aspects of deprescribing, as described above.

In each round, an online survey will be disseminated via email that prompts the panel members to review the statements provided based on their best judgment and experience. The response to each proposed recommendation or statement in the survey will be binary, either agree or disagree, with an optional free-text comment section at the end of each drug class section. This is so we can capture any valuable insights that are not in the statements provided. We will not consider a round valid if the response rate falls below 70%, as it may give rise to non-response bias [59]. Survey responses will remain confidential and accessible only to the guideline steering committee responsible for data analysis. Participants will complete the survey independently, without direct interaction with other survey respondents.

The participants will initially be given 14 days to complete the survey. The survey will be designed to automatically save responses, enabling participants to resume and complete the survey at a later time, even if they exit the survey before submitting. If participants are unable to complete the survey by the original deadline, individual extensions will be granted for a reasonable duration to accommodate their schedules. To further maximise the retention rate, we will provide an email update to all participants about the study progress, including the anticipated date for the next survey round, so the participants are prepared [60]. After each round is concluded, we will summarise and anonymise the feedback. We will share a brief feedback report that summarises the response percentages for each question to the panel members who participated, and they will be thanked for their contribution to the study.

##### Definition of consensus

In theory, consensus is achieved when all panel members agree or disagree on the items. However, a full agreement is rarely achieved and likely not feasible in a Delphi study. The goal of consensus is to reach a mutually acceptable level of agreement. Although consensus is fundamental to Delphi studies, a systematic review revealed that it is often poorly defined and rarely reported [61].It is recommended to pre-specify a threshold percentage for agreement [61].In this study, we will define 75% or greater agreement as consensus, as this percentage of agreement is generally considered acceptable in literature [40, 61, 62].

##### Delphi rounds

We will conduct at least one survey round to allow feedback and revision of responses. The most common number of rounds to reach consensus in practice is typically two to three [63]. Statements where consensus has been achieved will be incorporated into clinical practice guidelines as consensus-based recommendations. If consensus has not been achieved, the statements will be presented to the panel members in the next round. The steering committee will modify the survey in the next round to include refined statements to capture the evolving consensus. If consensus is still not achieved after a reasonable number of rounds, we will identify these items in the clinical practice guideline under ‘Areas of Debate’, highlighting a lack of consensus for future research. In this regard, no recommendation will be made.

##### Data analysis

We will collect information about the participants’ demographics, including name, email address, age, gender, and where relevant, the geographic location of their current primary work location, job title and number of years of experience. These data will be analysed descriptively [64]. As the focus of this study is on quantitative data collection and analyses for binary responses, the participants’ responses for each statement will be aggregated and analysed in percentages to determine consensus. We anticipate optional free-text comments may include main insights, reasons for agreement or disagreement, and any suggestions for statement revisions. Qualitative data collected from the free-text comments will be analysed thematically to identify common themes and topics that emerge from the participants’ responses.

### Patient and public involvement

#### Independent review

Following Phases 1 and 2, we will invite at least two independent expert peer-reviewers who are not part of the guideline development group to review the guideline using the AGREE-II instrument. The independent review stage helps identify areas for improvement before the guidelines are finalised by assessing the methodological quality of guidelines against the AGREE II instrument. The independent reviewers will be identified by the guideline development group through existing networks. They will have the expertise and experience relevant to the content of the guideline as well as an understanding of the context in which the guideline will be implemented.

#### Public consultation

Prior to finalising the overall guideline, we will also conduct a public consultation process to seek input from the wider community on the draft recommendations. This will ensure that the guideline recommendations are aligned with the community's values and expectations. The public includes individuals, patient organisations, policymakers, and professional organisations that will be involved in, or affected by, the implementation of the clinical recommendations of the guideline. We will notify the public of the opportunity to review the draft and share their written feedback via newspaper, emails, social media, website notices, or directly emailing relevant stakeholders. As part of the public consultation process, we will make the draft guideline available for a minimum period of 30 days on an online platform for public access. An extension of the consultation period may be considered if reasonable requests have been made from the public. Following the conclusion of the public consultation, we will prepare a public consultation report with a summary of the process and the changes made to the guidelines as a result. All stakeholders who have made a submission will be formally acknowledged in the guideline, provided they have given consent to do so.

## Discussion

Deprescribing is widely recognised as an essential aspect of good prescribing practice. Like any healthcare intervention, deprescribing is not without potential risks. Deprescribing may result in adverse drug withdrawal events, recurrence of symptoms, or worsening of the underlying condition for which the medicine was originally prescribed [49, 65]. For example, deprescribing antihypertensives may result in increased systolic blood pressure and worsened overall blood pressure control [66]. However, when conducted as a planned and supervised process, the risks associated with deprescribing can be minimised. Key strategies include thoughtful planning, shared decision-making, gradual dose tapering, and appropriate clinical monitoring [67]. The guideline development process will explicitly address potential harms and incorporate strategies for monitoring and risk mitigation across drug classes, drawing on findings from the systematic review and expert clinical consensus. Recommendations will provide practical guidance on tapering, monitoring, and, where necessary, reinitiating therapy. The guideline will emphasise the importance of obtaining informed consent and encouraging patients or carers to report any emerging symptoms following deprescribing. Clinicians will also be advised to document the rationale for deprescribing, the monitoring plan, and any adverse outcomes in the medical record.

The proposed approach discussed in this protocol is adapted based on several important considerations on the practical, operational, and resource issues while aiming to align with established guideline development frameworks to ensure methodological rigour and enhance credibility. The currently proposed guidelines present a large scope of targeted medicines. The option of including a systematic review and meta-analysis for each drug class was given deliberate and extensive consideration, and it was concluded that the length of time and resources rendered it inappropriate for the goal of this work. Instead, a comprehensive systematic review and meta-analysis were chosen that included evidence on deprescribing with broad inclusion criteria as detailed above. Given deprescribing is an emerging area and the limited availability of evidence for some drug classes, expert consensus and input from patient representatives offer a valuable alternative for recommendation development.

The final guideline will provide broad guidance for deprescribing common medicines used in older people, that complements existing single drug-class deprescribing guidelines and other treatment guidelines. The dissemination plan for the clinical practice guideline will be informed by the NHMRC approach and our ongoing knowledge of translation activities [68]. To ensure wide uptake across diverse healthcare settings, various channels will be used to disseminate the guideline including digital platforms (e.g. media release, social media channels, professional forums), professional networks (e.g. relevant societies, organisations, key target groups, and charities), and events (e.g. presentation at academic conferences, webinar). An impact log will be used to keep a record of the dissemination across various channels and to accumulate feedback using Google Analytics, Scopus, SciVal, Almetric Explorer, and Web of Science as appropriate. We anticipate the guideline to be implemented in a range of workplace settings, including but not limited to primary care clinics, hospitals, and residential aged care facilities where older people are cared for.

This study has limitations which relate to the scope of the guideline. We used PBS data to identify priority medicines where this guideline is most likely to have the greatest impact on clinical practice. However, a key limitation of using PBS data is that it does not capture medicines obtained without a prescription, such as over-the-counter or complementary medicines, or those dispensed through private prescriptions. As a result, despite our best efforts to ensure comprehensive coverage, some medicines will be excluded from the scope of this guideline. Additionally, for pragmatic reasons outlined earlier, we will rely on a single comprehensive systematic review to inform our recommendations across multiple drug classes. We acknowledge that this approach may not capture all relevant aspects of deprescribing for each medication. However, we anticipate that good practice statements will help facilitate the implementation of the recommendations. With additional resources in the future, subsequent iterations of the guideline may incorporate broader sources of evidence and further strengthen the recommendations.

## Data Availability

The datasets used and/or analysed during the current study are available from the corresponding author on reasonable request.

## Abbreviations

AGREE II: Appraisal of Guidelines for Research and Evaluation II
CINAHL: Cumulated Index to Nursing and Allied Health Literature
GRADE: Grading of Recommendations Assessment, Development, and Evaluation
NHMRC: National Health and Medical Research Council
PBS: Pharmaceutical Benefits Scheme
WHO: World Health Organisation
